# Cecum perforation in intestinal malrotation setting in a patient with chromosome 12p deletion syndrome: A case report

**DOI:** 10.1016/j.ijscr.2019.12.017

**Published:** 2019-12-17

**Authors:** João T. Oliveira, Paula Marques, J.M. Preza Fernandes, Tânia Teixeira, Marisa D. Santos, Ana Povo, Eurico Castro Alves

**Affiliations:** a Departamento de Cirurgia, Centro Hospitalar e Universitário do Porto, Porto, Portugal; b Departamento de Cirurgia, Instituto de Ciências Biomédicas Abel Salazar Universidade do Porto, Porto, Portugal; c Unidade de Cirurgia Colorretal, Centro Hospitalar e Universitário do Porto, Porto, Portugal

**Keywords:** Intestinal malrotation, Intestinal obstruction, Syndrome, Case report

## Abstract

**Introduction:**

Intestinal malrotation results from failure of the normal gut rotation during embryological development. It is usually diagnosed in early childhood when it becomes symptomatic. Aetiology of intestinal malrotation has been scarcely addressed although relevant roles have been attributed to a few genes involved in gastrointestinal formation and association with certain syndromes has been suggested.

**Presentation of case:**

We describe the case of a 23-year-old woman with 12p deletion syndrome who presented with clinical symptoms of occlusion to the emergency department. Analytically, an elevation of inflammatory parameters was confirmed and imaging revealed pneumoperitoneum originated on cecum perforation. The patient was submitted to surgery with favorable evolution.

**Discussion:**

Clinical manifestation of intestinal malrotation is uncommon in the adult population but can have severe consequences if not diagnosed early. The abnormal positioning of the duodenojejunal loop compressed by Ladd’s bands, can lead to obstruction and ischemia. Surgery via Ladd’s procedure commonly applies and elective treatment may prevent added morbidity. Intestinal malrotation has been associated to certain syndromes but no prior association to chromosome 12p deletion has been described. Occlusion in a patient with 12p chromosome deletion should raise prompt suspicion for intestinal malrotation. Moreover, diagnosis of 12p chromosome deletion should increase attention towards gastrointestinal changes since elective surgery may diminish morbidity.

**Conclusion:**

Intestinal malrotation results from abnormal embryological rotation of the midgut and is associated with certain syndromes. This paper firstly associates intestinal malrotation to chromosome 12p deletion. The possibility to address it electively may prevent morbidity in patients with this syndrome.

## Highlights

• Intestinal malrotation is an uncommon diagnosis that can be associated with genetic syndromes.
• Intestinal malrotation presenting as abdominal pain in an adult patient with chromosome 12p deletion syndrome is herein described.
• Early diagnosis allows surgical planning obviating the need for enterectomy and ostomy.

## 1 Introduction

Intestinal malrotation results from a disturbance in the normal rotation of the embryonic gut during early development. Yearly incidences between 0,2%–1% have been reported [1].

The majority of intestinal malrotation diagnosis is usually performed in early childhood (90%), although several papers have described relevant numbers on diagnosis made in young adults [1,2].

Few efforts have been conducted to determine the aetiology of this disturbance. Nonetheless, it has been shown that several occur in association with syndromic forms and, moreover, relevant roles have been attributed to Foxf1, Irx3, Pitx2, Isl 1 and Nodal in terms of genetic landscape [2].

The majority of adults with symptomatic intestinal malrotation present with clinical features that may include vomiting, abdominal pain and diarrhoea, although other symptoms can be present [3]. A small percentage (10–15%) may present with more serious conditions such as midgut volvulus.

The diagnosis of intestinal malrotation relies mostly on the suggestive clinical symptoms alongside with concordant imaging analysis, and for the latter, a CT scan constitutes a valuable tool. The gold standard treatment is surgery, but this may be adjusted in terms of timing depending on the acuteness of the symptoms [4].

Malrotation has been linked to certain pathologies and syndromes such as Martinez-Frias or the Megacystis, Microcolon and Intestinal Hypoperistalsis (MMIH) syndromes [2,5,6].

In this paper, we describe the case of a 23-year-old woman with 12p deletion syndrome who presented with occlusion due to a midgut volvulus.

To our knowledge, no prior description of chromosome 12p deletion and intestinal malrotation has been made in the literature.

This work has been reported in line with the SCARE criteria [7].

## 2 Presentation of case

A 23-year old woman was admitted to the emergency department with vomiting, anorexia, prostration and fever lasting for 4 days. She has a deletion of chromosome 12 short arm. Alterations of the intestinal habit were not described, having the patient one dejection the day before observation.

Clinically, the patient presented a distended abdomen with diffuse tympanism, with a pain expression associated with palpation and peritoneal irritation signs.

**Fig. 1:**
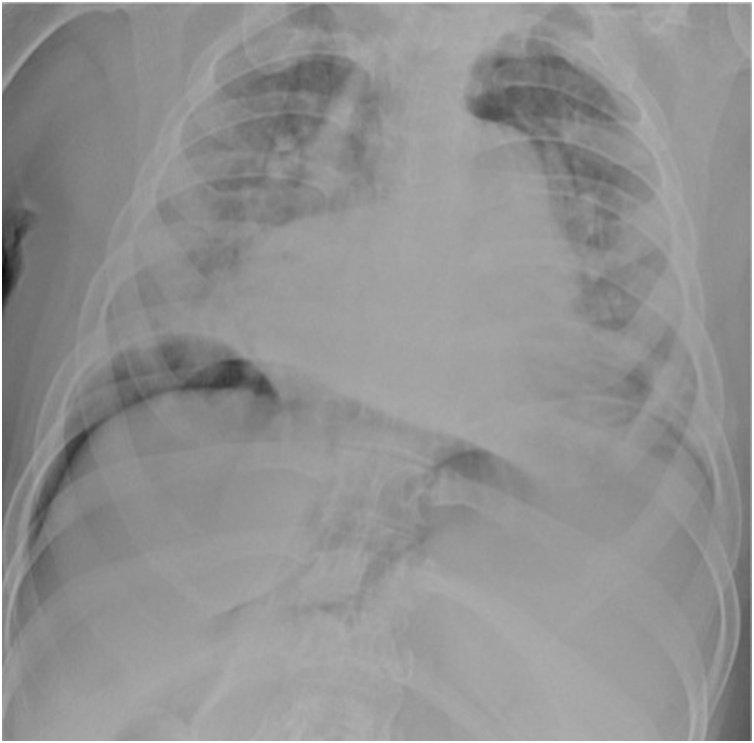
Pneumoperitoneum evidenced on abdominal radiograph.

**Fig. 2:**
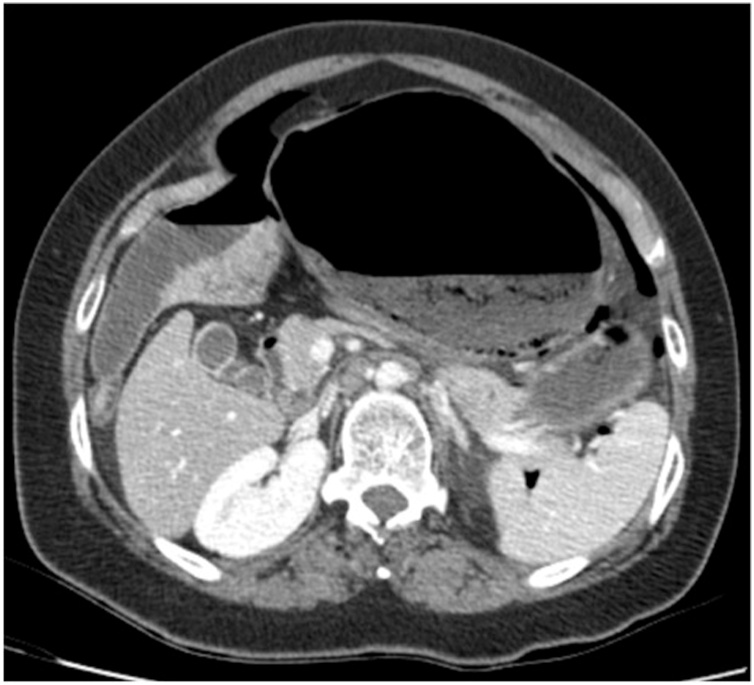
Abdominal CT scan showing a grossly distended cecum in the upper left quadrant.

**Fig. 3:**
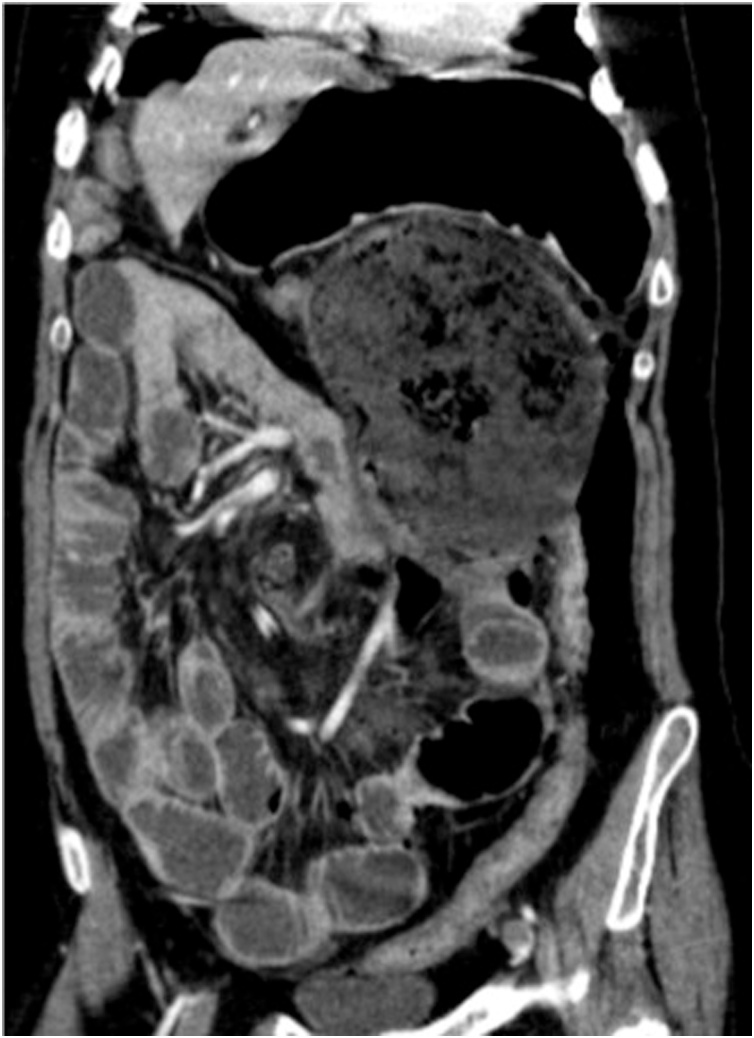
Abdominal CT scan evidencing intestinal malrotation with high intestinal obstruction due to compression of the stomach by the cecum.

The workup study showed elevation of white blood cells and C-reactive protein and the abdominal radiograph showed bilateral subdiaphragmatic free gas (Fig. 1). CT scan presented medium volume pneumoperitoneum, high intestinal obstruction due to compression of the stomach caused by the cecum, intestinal malrotation with the jejunum right to the midline, superior mesenteric artery deviated also to the right following the jejunum and cecum in a markedly anomalous position, rotated and twisted over itself, conditioning extrinsic compression of the stomach with the remaining colon collapsed, posteriorly to the cecum (Fig. 2, Fig. 3).

**Fig. 4:**
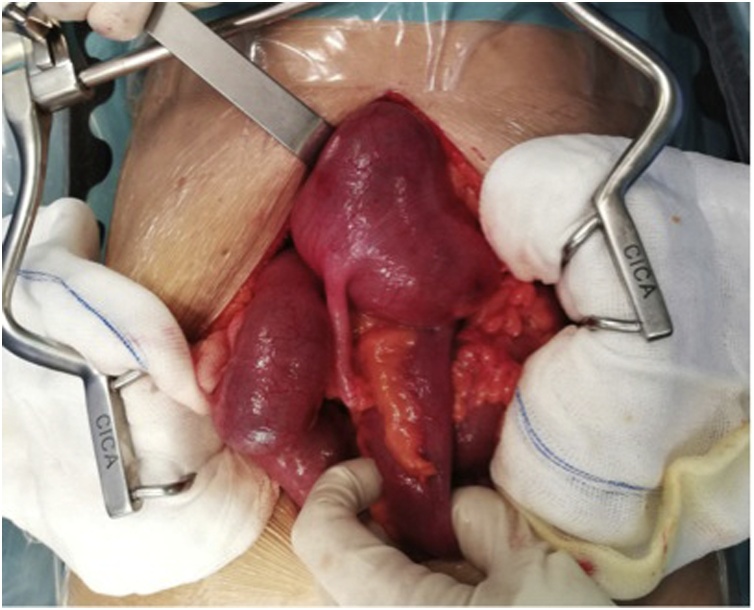
Exploratory laparotomy evidencing a distended cecum with 20 cm diameter.

**Fig. 5:**
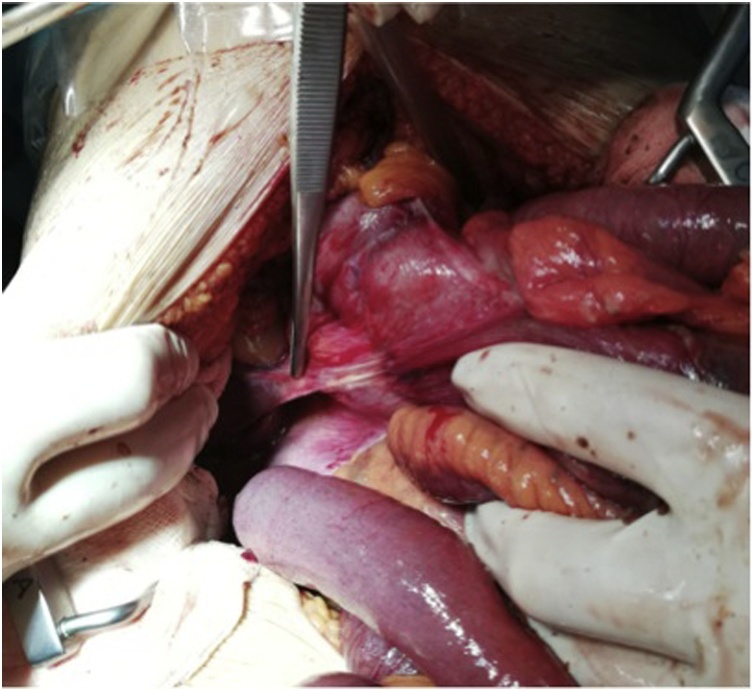
Closed loop obstruction filliated in intestinal malrotation with a Ladd band (indicated by the tip of the forceps).

Since there was a perforation due to a midgut volvulus, an exploratory laparotomy was performed. Intra-operatively, besides confirming the CT scan alterations with a diameter of the cecum above 20 cm (Fig. 4), two punctiform areas of perforation were identified, compatible with a close loop obstruction filliated in a malrotation syndrome with a Ladd band (Fig. 5).

Ligation of the Ladd band followed by an ileocecal resection was performed, with a latero-lateral ileocolic anastomosis. The patient was then admitted in the Intensive Care Unit due to respiratory insufficiency assumed as an aspiration pneumonitis.

The respiratory insufficiency was resolved under empiric antibiotic therapy and the patient was admitted to the surgical ward on the 8th post-operative day.

On the 9th post-operative day, the patient required a second operation due to an evisceration. Intra-operatively findings compatible with a small abscess adjacent to the anastomosis and evisceration caused by aponeurotic sheet necrosis were found. Orientation of the abscess with a perianastomotic drain and closure of aponeuroses were performed, reinforced with transparietal sutures.

The patient presented favorable clinical evolution, with maintained intestinal habit and good tolerance to reintroduction of diet. Due to isolation of *Candida albicans* in the abscess pus, a 7-day antifungal course was completed, with good response. Clinical discharge occurred on the 19th post-operative day.

## 3 Discussion

Intestinal malrotation is the result of the failure of the normal anti-clockwise rotation of the midgut along its vascular pedicle as it returns from herniation from the umbilical cord in the 5th–12th week of embryological development [6].

This change in normal development conditions an abnormal positioning of the duodenojejunal loop compressed by Ladd’s bands (fibrous peritoneal tissue that attach the cecum to the retroperitoneum), causing obstruction of the duodenum and the narrow fixation of the base of the mesentery, increasing the risk of superior mesenteric artery ischemia [1,4].

Presentation of intestinal malrotation in adults tends to follow a more chronic installation, with unspecific symptoms such as abdominal pain, intermittent alternate diarrhoea and constipation and malabsorption. Clinical scenarios that involve sudden onset abdominal pain and vomiting are acute and generally related to midgut volvulus.

In this paper, we describe the case of a 23-year-old female with intestinal malrotation that presented acutely in an emergency setting with symptoms of vomiting and abdominal distension. Intraoperatively, these symptoms were confirmed to be associated with rotation of the cecum. This case in the adult has an unusual acute presentation and has not presented at early age, which is less common based on literature findings [8,9].

Given the critical onset of symptoms and condition of the patient, surgery was the following step. The outcomes revealed successful in agreement with prior publications [10].

Based on the literature, the positive evolution of this patient places it in a more limited number of satisfactory outputs, since mortality for intestinal malrotation following surgery ranges from 0 to 25% in adult patients, and those who present with acute midgut volvulus carry the highest mortality rates [3].

Descriptions of association of intestinal malrotation with syndromes such as Martinez-Frias or the Megacystis, Microcolon and Intestinal Hypoperistalsis (MMIH) syndromes have been made. However, no prior description of chromosome 12p deletion and intestinal malrotation exists to our knowledge.

We have searched the OMIM database for gene modifications/rearrangements known to be associated with 12p deletion and compared them with genes that have proven association to gut normal development, such as Foxf1, Irx3, Pitx2, Isl 1 and Nodal [2]. The deletion of 12p chromosome does not directly affect any of these genes, suggesting that potential genetic changes related with abnormal intestinal development in this syndrome might be linked to other genetic profiles or be the result of multifactorial events unknown at this stage. Although representing the minority of cases, some studies in the literature describe intestinal malrotation in a considerable number of congenital malformations [1].

Based on these observations, and on the work herein described, the observation of acute intestinal occlusion in a patient with 12p chromosome deletion should convey prompt suspicion for intestinal torsion related to midgut volvulus, due to the presence of an intestinal malrotation.

## 4 Conclusion

Intestinal malrotation results from a failure in normal rotation of the midgut during the early stages of embryological development. It is far more often diagnosed during childhood than in the adult. In the latter, it presents in a more indolent form, with either subacute or chronic symptons. An acute setting as described herein is uncommon. Intestinal malrotation is associated to certain syndromes and, as detailed in this clinical case, it can be present in patients carrying chromosome 12p deletion. This work, in accordance with others described in the literature, should increase awareness to the relationship between certain syndromes and gastrointestinal malformations since an elective surgical treatment of these patients as the ability to mitigate some of the morbidities with surgical procedures conducted in an emergency setting.

## Funding

We have not received any funding for this research.

## Ethical approval

Ethical approval was not obtained from the institution as the article is a complete de-identified case report and no experimentation was performed.

## Consent

Written informed consent was obtained from the caregiver/legal representative for publication of this case report and accompanying images.

## Registration of research studies

This is a retrospective observational case report. It is the authors opinion that registry does not apply to this case.

## Guarantor

Dr João Oliveira, Drª Paula Marques, Dr JM Preza Fernandes, Drª Tânia Teixeira, Profª Drª Marisa Santos, Profª Drª Ana Povo, Dr Eurico Castro Alves.

## Provenance and peer review

Not commissioned, externally peer-reviewed.

## CRediT authorship contribution statement

**João T. Oliveira:** Investigation, Methodology, Writing - original draft, Writing - review & editing. **Paula Marques:** Investigation, Methodology, Writing - original draft, Writing - review & editing. **J.M. Preza Fernandes:** Investigation, Methodology, Writing - review & editing. **Tânia Teixeira:** Writing - review & editing. **Marisa D. Santos:** Writing - review & editing. **Ana Povo:** Investigation, Methodology, Writing - review & editing. **Eurico Castro Alves:** Writing - review & editing.

## Declaration of Competing Interest

There are no conflicts of interest to declare.
